# COVID-19 drives medical education reform to promote “healthy China 2030” action plan

**DOI:** 10.3389/fpubh.2024.1465781

**Published:** 2024-10-25

**Authors:** Liping Han, Fuyun Wu

**Affiliations:** ^1^School of Humanities and Social Science, Hubei University of Medicine, Shiyan, China; ^2^School of Basic Medical Sciences, Hubei University of Medicine, Shiyan, China

**Keywords:** COVID-19, public health, medical education, talent cultivation, healthy China 2030

## Abstract

In June 2019, the Chinese government proposed the Healthy China Action Plan (2019–2030) development strategy, which focuses on disease prevention and health promotion. It is expected that by 2030, the national health literacy level will be significantly improved, premature mortality caused by major chronic diseases will be significantly reduced, the average healthy life expectancy will be greatly improved, and the main health indicators of residents will enter the ranks of high-income countries. Unfortunately, at the end of 2019, COVID-19 began to break out in Wuhan, China, which had a huge impact on China's economy and people's health. A series of problems in China's health care and medical education were exposed in the prevention and treatment of the epidemic. How to reform medical education and build a medical talent training system with Chinese characteristics is the key to achieving China's Health 2030 strategy. This article will explore the direction of medical education reform in China under the background of the “Healthy China 2030” strategy and the post pandemic era.

## 1 Introduction

“Healthy China 2030” is an important strategic plan proposed by China, aimed at promoting the construction of a healthy China, improving the health level of the entire population, and promoting sustainable economic and social development (1). It is expected that by 2025, China's health system will become more perfect, the ability to prevent and respond to major epidemics and public health emergencies will be significantly enhanced, the innovation ability of health technology will be significantly strengthened, and the average life expectancy of health will increase proportionally (2–4). The implementation of this strategy coincides with the outbreak of the COVID-19, which exposed the weaknesses of public health system and current medical education in China (5–7). Therefore, the Chinese government and educational institutions have introduced a series of measures to address these issues. After years of education reform, the training mode, educational philosophy, and innovative practical ability of medical students have undergone significant changes. However, for responding to sudden public safety and health emergencies, achieving the national health strategy, and cultivating medical talents with both modern medical technology and innovation capabilities, medical education reform still needs to be continuously explored.

### 1.1 Impact of COVID-19 on Chinese medical education

The outbreak of the COVID-19 epidemic has undoubtedly exposed some weaknesses in China's medical education model. Firstly, the training of emergency response ability is insufficient. Medical education often focuses more on the diagnosis and treatment of routine diseases in daily life, with relatively weak training in emergency response to public health emergencies. At the beginning of the COVID-19 epidemic, many medical students and medical staff showed a lack of experience and strategies to deal with it (8). Secondly, the integration of interdisciplinary education is not deep enough. The response to the COVID-19 epidemic requires multidisciplinary knowledge and skills, such as epidemiology, statistics, sociology, psychology, etc. However, in the traditional medical education model, the boundaries between disciplines are relatively clear, and interdisciplinary integration education has not been fully developed, resulting in difficulties in achieving interdisciplinary collaboration in epidemic prevention and control (9). Furthermore, the preparation of online education resources and platforms is insufficient. During the epidemic, offline teaching was restricted and forced to switch to online teaching on a large scale. However, some medical education institutions lack in the construction of online education resources and the adaptability of teachers and students to online teaching, which affects the teaching effectiveness and quality (10). Finally, professional ethics and humanistic care education need to be strengthened. Some studies have investigated the humanistic care ability of Chinese medical staff, but the results are not satisfactory (11, 12). The existing medical education may not have sufficient training in this area, resulting in some medical staff experiencing difficulties in psychological adjustment or insufficient care for patients when facing enormous pressure and difficulties. A cross sectional study showed that during the COVID-19, medical staff in Wuhan experienced tremendous mental pressure and serious mental health problems. The mental state of medical staff was directly related to the patient's nursing experience (13, 14).

## 2 Medical education reform in the context of the “Healthy China 2030” strategy

The implementation of the Healthy China strategy is the foundation for achieving the Chinese Dream of rejuvenation of the Chinese nation. The development of medical education in China is currently at a special historical juncture. It is necessary to consider the future of medical education and clarify the direction of medical education reform to better cope with new challenges.

### 2.1 Reforming teaching methods to promote digitalization of education

**Figure 1:**
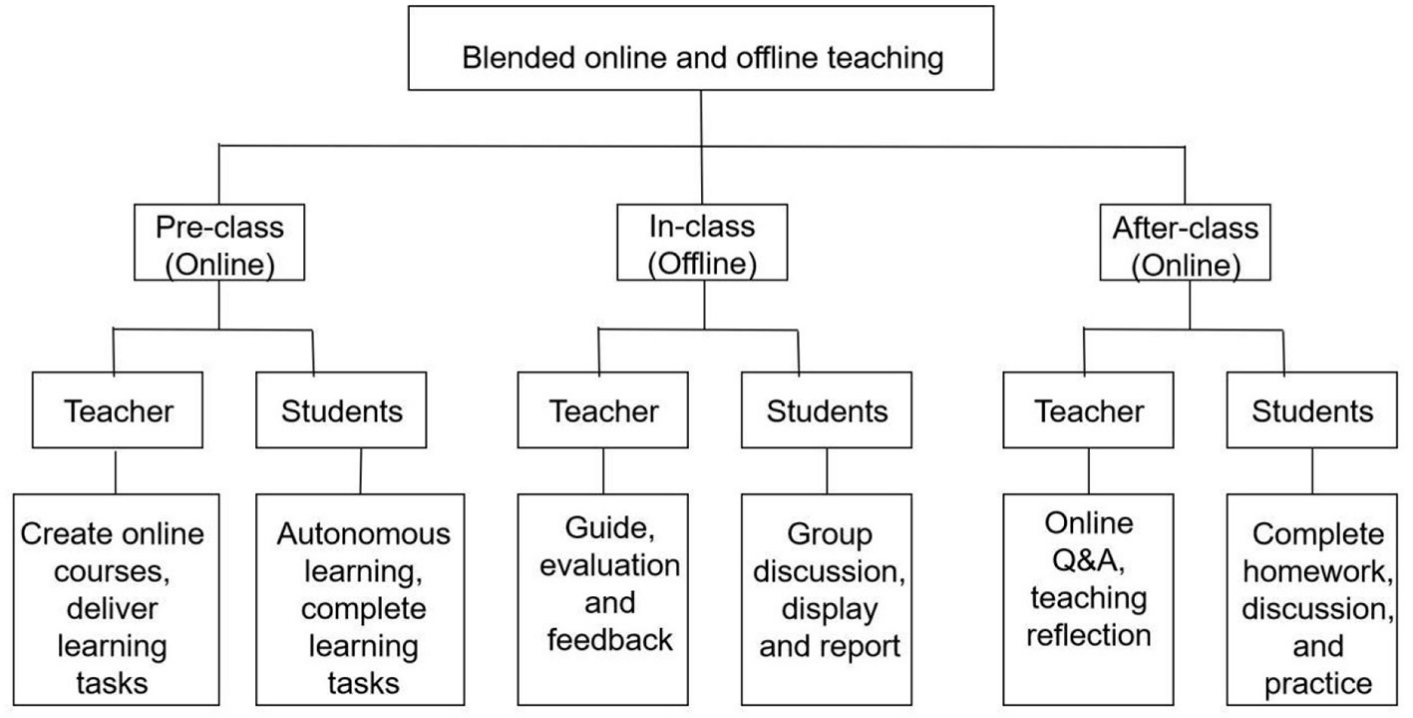
Blended online and offline teaching mode.

During the COVID-19 pandemic, offline teaching was limited, which prompted the rapid development of online teaching in medical education. Major medical schools have set up online teaching platforms, conducting live courses, recorded courses, and online interactive discussions. This not only breaks the limitations of time and space, allowing students to access learning resources anytime and anywhere, but also cultivates their ability to learn independently. Through years of development, the blended learning model of online and offline has been greatly promoted (Figure 1).

#### 2.1.1 Challenges of blended learning mode

The blended learning model of online and offline is the integration of traditional classroom teaching in offline and learning in online network environment. With the development of information technology, products related to education such as MOOCs, micro courses, various learning apps, and mobile learning platforms have emerged. While effectively expanding educational time and space, their advantages such as flexibility, accuracy, richness, fun, and personalization have also been widely recognized by educators and learners. Students transition from passive learning to active learning and from individual learning to team collaboration through online resource previews, extracurricular assignments, group case discussions, and other teaching activities (15–17). At the same time, online teaching has also promoted innovation in educational technology, such as the application of virtual laboratories, simulated clinical scenarios, etc. However, the blended learning model of online and offline also faces many challenges. It should follow the law of knowledge dissemination and the students' development. Teachers need to make full use of internet technology to carry out the integrated design of online and offline teaching, rather than a simple mechanical combination of online and offline teaching content and teaching mode. The design of integrated teaching mode needs to fully consider the cognitive level of students, the progressive nature of teaching content, the class hour allocation and classroom activities of online and offline teaching. In addition, teachers should also make full use of the network platform and internet technology after class to understand students' online learning data, summarize students' online learning, and give responses in offline face-to-face teaching to optimize online teaching design (18, 19).

#### 2.1.2 The development of digital education in China

In addition, information technology should be deeply integrated with curriculum teaching to promote digitalization of education. Modern information technology is the core force driving curriculum and teaching reform (20). By utilizing its fast transmission speed and strong information processing capabilities, high-quality and diverse curriculum resources are categorized and aggregated in specialized cyberspace or designated cloud platforms to solve the problem of course resource sharing. Secondly, utilizing cloud computing and cloud services, big data analysis, human-computer interaction and other technologies to record the learning process, perceive learning status, and conduct real-time statistics and analysis, intelligent learning guidance and assistance are provided for students' learning. At present, China's digital education is developing rapidly. By the end of 2023, 519,000 educational institutions across China had linked to the national smart education platform, benefiting 18.8 million teachers and 293 million learners. It had over 100 million registered users from 200 countries, with 36.7 billion visits. The platform has incorporated a vast treasure trove of digital resources, including 27,000 university courses, and nearly 500 courses on innovation and entrepreneurship, effectively improving students' innovation and knowledge transfer abilities (21). China's modern higher education also cannot do without cooperation with countries around the world. The internationalization of digital education is an important trend in the development of education, which has also brought new opportunities and challenges to Chinese education. China still needs to further promote the construction of a global digital education cooperation platform and promote the exchange and integration of educational resources, educational models and educational concepts through digital technology and internet platforms worldwide.

### 2.2 Optimize the system of medical talent cultivation and promote access to medical innovation

**Figure 2:**
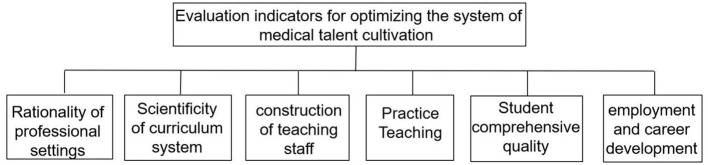
Evaluation indicators for optimizing the system of medical talent cultivation.

The COVID-19 has exposed a series of problems in China's medical talent training system, which are mainly manifested in the large enrollment scale of medical education, but the overall level is low (22). The current hierarchical structure and quality level of medical talents are not in line with the development of the economy and society. There is a significant shortage of general practitioners and high-level public health talents (23, 24). Therefore, optimizing the system of medical talent cultivation and accelerating the development of medical innovation are crucial for the “Healthy China 2030” strategy. In addition, it is necessary to establish relevant evaluation indicators to guide and promote medical education reform and scientific research innovation development (Figure 2). In terms of medical talent cultivation, it can be evaluated from multiple dimensions such as the rationality of professional settings, the rationality of hierarchical structure, the construction of teaching staff, and employment and career development. For example, examining whether the professional settings are in line with the needs of the health industry, whether the proportion of medical talents at different levels is reasonable, whether students' clinical skills, research and innovation abilities, and humanistic literacy levels have been effectively improved, whether the teaching staff has rich teaching experience and professional literacy, and what the employment rate and career development prospects of graduates are. In terms of medical innovation development, it can be evaluated from the aspects of scientific research investment, scientific research output, technology transformation efficiency, and innovative enterprise development.

#### 2.2.1 Strengthen the training of general practitioners

At present, the number of general practitioners in China is < 9% of the total number of clinical doctors, and the education level of general practitioners is low, with insufficient ability to diagnose, treat, and prevent diseases (25). High level composite specialist doctors, including professionals in infectious diseases, respiratory diseases, public health, etc., are lacking, and their ability to respond to major outbreaks of infectious diseases is weak. The Chinese government should increase its efforts in cultivating comprehensive medical talents, gradually expand the scale of targeted free comprehensive medical student training for grassroots services. At the same time, educational institutions should systematically plan the general practice teaching system, strengthen general practice education for all medical students, establish demonstration bases for general practice teaching, accelerate the reform of the general practitioner salary system, and expand the career development prospects of general practitioners.

#### 2.2.2 Strengthen the construction of public health service system

China need to accelerate the construction of a high-level public health talent training system, establish a number of high-level public health colleges. However, the teaching, research, and practical implementation functions of China's public health system are divided between public health colleges and disease prevention and control centers, resulting in a separation between the development of public health theory and practice. Therefore, it is necessary to strengthen practical teaching and enhance the cooperation between medical colleges, disease prevention and control centers, and infectious disease hospitals in medical research and teaching. Through practical teaching, such as clinical internships, public health internships, etc., medical students can apply theoretical knowledge to practical work, improve their practical and problem-solving abilities (26). Medical education institutions should also collaborate with public health institutions in scientific research, cultivating students' research abilities and innovative thinking, enabling them to have the ability to prevent and control infectious diseases, as well as comprehensive qualities to respond to public health emergencies (27).

#### 2.2.3 Promote the inheritance, innovation, and development of traditional Chinese medicine

Traditional Chinese medicine has played a unique role in the prevention and control of the COVID-19, and demonstrated the value and role of traditional Chinese medicine in responding to major public health events (28). Similarly, traditional Chinese medicine plays a very important role in China's health strategy. It has significant advantages in disease prevention and chronic disease management (29). Therefore, traditional Chinese medicine plays an irreplaceable role in China's health strategy. However, for a long time, there have been problems and bottlenecks in the cultivation of traditional Chinese medicine talents, such as long cycles, insufficient medical innovation, and prevention and treatment capabilities for major diseases. Therefore, the cultivation of traditional Chinese medicine talents needs to follow the basic laws of the development of traditional Chinese medicine, establish a thinking mode with Chinese medicine characteristics, and organically integrate traditional Chinese medicine disciplines with interdisciplinary fields. It is also necessary to comprehensively apply new technologies and methods such as artificial intelligence to meet the needs of modern medical development and public health.

#### 2.2.4 Strengthen the cultivation of interdisciplinary talents in medicine

Multidisciplinary integration has become a trend in global medical research and healthcare services. With the aging population and changes in lifestyle, diseases are becoming increasingly complex and diverse. To cultivate interdisciplinary talents who can integrate knowledge and methods from different disciplines, conduct in-depth research on the pathogenesis, diagnosis, and treatment of diseases from multiple perspectives, and provide more comprehensive and effective solutions for solving complex diseases. Secondly, modern healthcare is shifting from disease centered to health centered. This requires the intersection of medicine with public health, environmental science, nutrition, and other disciplines, focusing on disease prevention and health promotion, and achieving full life cycle health management (30). In 2024, the Chinese Ministry of Education added five “cross fusion” majors to meet major national needs and serve the new development trend of the health industry. These 5 majors are: Medical Device and Equipment Engineering, Geriatric Medicine and Health, Health and Medical Security, Pharmaceutical Economics and Management, and Biomedical Data Science. These majors promote the cross disciplinary integration of medical engineering, medical science, and medical humanities, which is conducive to the cultivation of comprehensive talents. However, new requirements have been put forward for the reform of curriculum, teaching, faculty, and teaching evaluation.

### 2.3 Improve the quality of medical student training and cultivate innovative medical talents

High quality medical talents are an important foundation and guarantee for building a healthy China. How to better cultivate high-level and top-notch medical innovation talents that meet the needs of the new era? How to make Chinese medical talents have stronger international competitiveness? These are the major issues currently facing the reform of medical education in China.

#### 2.3.1 Humanistic education for medical students

Medical students should possess good humanistic qualities. Having humanistic literacy can enable medical students to better understand patients' psychological, emotional, and social backgrounds, thereby establishing a relationship of trust and respect, improving patient compliance and treatment effectiveness (31). At present, many medical colleges in China still adhere to the traditional concept that doctors only treat diseases and save lives, and only focus on imparting professional knowledge, neglecting the education of humanities and the cultivation of students' comprehensive qualities. But the modern medical model is shifting from a simple biomedical model to a biopsychosocial medical model, requiring medical students to have the ability to comprehensively consider patients' physiological, psychological, and social factors. Humanistic literacy is the key to achieving this transformation. Therefore, in order to improve the level of humanistic education for medical students, schools must incorporate humanistic courses into the curriculum system and make them reach a certain proportion and offer more public elective courses in humanities to enhance students' cultural literacy. In addition, clinical reception and doctor-patient communication skills training courses should be offered to enable students to systematically learn doctor-patient communication skills. The volunteer practical activities should be carried out to improve the professional ethics of medical students (32).

#### 2.3.2 Leadership in medical education

With the increasing complexity of the healthcare system, the continuous advancement of medical technology, and the diversification of patient needs, the healthcare industry urgently needs talents with strong leadership to cope with various challenges. Medical professionals with leadership skills can effectively coordinate medical teams, optimize the allocation of medical resources, promote interdisciplinary cooperation, drive the development of medical research and education, and thus enhance the overall level of the medical industry (33, 34). So, how to cultivate leadership in medical education? Firstly, it is necessary to provide students with comprehensive knowledge of leadership theory. Incorporate leadership related courses into the curriculum to help students understand the concepts, principles, and methods of leadership. Through teaching methods such as case analysis and group discussions, help students master effective leadership strategies and communication skills. Medical education should provide rich practical opportunities for students to exercise their leadership skills in a real medical environment. For example, organizing students to participate in the planning and implementation of medical projects, encouraging them to play a leadership role in the team, and solving practical problems.

#### 2.3.3 Scientific research and innovation in medical education

Medicine is a constantly evolving discipline, with new disease challenges and health needs constantly emerging. Medical students with research and innovation abilities can actively engage in medical research, explore unknown fields, and provide new ideas and methods for solving medical problems. They are able to use innovative thinking to optimize medical processes, introduce new technologies and methods, improve the accuracy of disease diagnosis, and treatment effectiveness. For the China's health strategy, the research and innovation capabilities of medical students are of great value in responding to major public health events. For example, innovative scientific research achievements in infectious disease prevention and control, chronic disease prevention and control, and emergency response to sudden health emergencies can provide scientific basis for policy formulation and measure implementation. In order to better cultivate the research and innovation abilities of medical students, the medical education system needs to increase research practice courses, provide more opportunities to participate in research projects, strengthen mentor guidance, establish interdisciplinary communication platforms, and create an academic atmosphere that encourages innovation (35, 36). At the same time, the government and society should increase investment in medical research, support innovative research by medical students, and promote the transformation and application of scientific research results.

#### 2.3.4 Mental health and medical education

In the process of medical education reform, the cultivation of mental health qualities among medical students plays a crucial and far-reaching role. The study of medical knowledge is arduous and complex, and clinical practice requires high precision and responsibility, which puts enormous academic pressure on medical students. Medical students with mental health have stronger resilience and emotional regulation abilities, and can cope with difficulties and challenges in learning with a positive attitude, thereby improving learning efficiency and quality (37, 38). Medical students with mental health are often more confident and empathetic, able to establish harmonious doctor-patient relationships and teamwork. To strengthen mental health education for medical students, the first step is to optimize the curriculum design. Incorporate mental health education into compulsory courses, systematically impart mental health knowledge and coping skills, so that medical students can self identify and deal with common psychological problems. At the same time, diversified teaching methods such as case analysis, group discussions, role-playing, etc. should be adopted to enhance the attractiveness and practicality of the course. Schools should establish specialized psychological counseling centers equipped with professional counselors to provide individual and group counseling services for medical students.

## 3 Conclusion

The COVID-19 epidemic has not only brought challenges to China's public health and medical education, but also brought new development opportunities. On the one hand, the epidemic has promoted the innovation of medical education model. The development of online teaching platforms and the abundance of digital teaching resources have provided a broader space and more diverse means for medical education, making personalized, and lifelong learning possible. On the other hand, the epidemic has prompted medical education to pay more attention to interdisciplinary training. The interdisciplinary integration of public health, clinical medicine, information technology, and other fields provides a higher quality training platform for new medical education, which will cultivate medical talents with comprehensive abilities and better serve China's health strategy. In the post pandemic era, the public's demand for health services is increasing day by day. However, compared with other countries, factors such as China's economic development level, population structure, socio-cultural background, and healthcare system result in certain differences in healthcare demand. The healthcare needs of economically developed countries are more diversified and high-end, including the demand for advanced medical technology, drugs, and medical services. China may place greater emphasis on the popularization and accessibility of basic healthcare. The aging population in China is gradually increasing, and the demand for long-term care, rehabilitation, and chronic disease management is growing. Sociocultural background can also affect people's perception of health and their demand for healthcare. China and some other Asian countries have a high demand for traditional Chinese medicine. The healthcare systems of different countries can also lead to differences in healthcare needs. China implements a universal medical insurance system, mainly to ensure the basic medical needs of residents. The demand for healthcare is increasingly showing characteristics of diversification, personalization, and high quality. New challenges and requirements have been put forward for medical education. Strengthening international exchanges and cooperation in the field of healthcare, drawing on advanced medical education systems and healthcare models from abroad, will help promote China's healthcare level. Under the Healthy China (2019–2030) strategy, the medical and health industry is entering a new stage of development. Medical education reform can cultivate more high-quality medical talents that meet the needs of the times, improve the overall quality and ability of the medical and health team, which will help strengthen the construction of the public health system, enhance the ability to respond to public health emergencies, improve the disease prevention and control system, promote fairness, accessibility, and quality improvement of medical services, thereby comprehensively and cyclically safeguarding people's health, effectively promoting the implementation of China's health strategy.

## Data Availability

The original contributions presented in the study are included in the article/supplementary material, further inquiries can be directed to the corresponding author.
